# CXCL13 antibody for the treatment of autoimmune disorders

**DOI:** 10.1186/s12865-015-0068-1

**Published:** 2015-02-12

**Authors:** Ekaterina Klimatcheva, Tracy Pandina, Christine Reilly, Sebold Torno, Holm Bussler, Maria Scrivens, Alan Jonason, Crystal Mallow, Michael Doherty, Mark Paris, Ernest S Smith, Maurice Zauderer

**Affiliations:** Vaccinex, Inc, 1895 Mt. Hope Avenue, Rochester, NY 14620 USA

**Keywords:** CXCL13, Chemokine, Monoclonal antibody, Collagen-induced arthritis, Experimental autoimmune encephalomyelitis

## Abstract

**Background:**

Homeostatic B Cell-Attracting chemokine 1 (BCA-1) otherwise known as CXCL13 is constitutively expressed in secondary lymphoid organs by follicular dendritic cells (FDC) and macrophages. It is the only known ligand for the CXCR5 receptor, which is expressed on mature B cells, follicular helper T cells (Tfh), Th17 cells and regulatory T (Treg) cells. Aberrant expression of CXCL13 within ectopic germinal centers has been linked to the development of autoimmune disorders (e.g. Rheumatoid Arthritis, Multiple Sclerosis, Systemic Lupus Erythematosis). We, therefore, hypothesized that antibody-mediated disruption of the CXCL13 signaling pathway would interfere with the formation of ectopic lymphoid follicles in the target organs and inhibit autoimmune disease progression. This work describes pre-clinical development of human anti-CXCL13 antibody MAb 5261 and includes therapeutic efficacy data of its mouse counterpart in murine models of autoimmunity.

**Results:**

We developed a human IgG1 monoclonal antibody, MAb 5261 that specifically binds to human, rodent and primate CXCL13 with an affinity of approximately 5 nM and is capable of neutralizing the activity of CXCL13 from these various species in *in vitro* functional assays. For *in vivo* studies we have engineered a chimeric antibody to contain the same human heavy and light chain variable genes along with mouse constant regions. Treatment with this antibody led to a reduction in the number of germinal centers in mice immunized with 4-Hydroxy-3-nitrophenylacetyl hapten conjugated to Keyhole Limpet Hemocyanin (NP-KLH) and, in adoptive transfer studies, interfered with the trafficking of B cells to the B cell areas of mouse spleen. Furthermore, this mouse anti-CXCL13 antibody demonstrated efficacy in a mouse model of Rheumatoid arthritis (Collagen-Induced Arthritis (CIA)) and Th17-mediated murine model of Multiple Sclerosis (passively-induced Experimental Autoimmune Encephalomyelitis (EAE)).

**Conclusions:**

We developed a novel therapeutic antibody targeting CXCL13-mediated signaling pathway for the treatment of autoimmune disorders.

## Background

Chemokines are small (7–15 kDa) inducible peptides involved in migration and retention of leukocytes in tissues under physiological and pathological conditions. The chemokines exert their effects through G-protein coupled receptors on target cells. Despite the potential pitfalls (promiscuity in chemokine/receptor interactions; multiple chemokine targets in any given pathologic conditions; broad receptor distribution), the chemokine system represents an attractive therapeutic target for a broad variety of autoimmune, inflammatory and oncology disorders and all components of the system can be potentially targeted by therapeutic agents.

The chemokine CXCL13 is constitutively expressed in secondary lymphoid organs (spleen, lymph nodes and Peyer’s patches) by FDC and macrophages [1,2]. CXCL13 primarily acts through G-protein-coupled CXCR5 receptor (Burkitt’s lymphoma receptor 1) expressed on mature B lymphocytes [3,4], CD4+ follicular helper T cells, (Tfh, antigen-primed Th cells [5,6], CD4+ Th17 cells [7], minor subset of CD8+ T cells and activated tonsil Treg cells [8,9]. CXCL13 has also been shown to interact along with the inflammatory chemokines CXCL9, CXCL10 and CXCL11, with the receptor CXCR3, albeit at lower affinity than with CXCR5. Expression of CXCR3 is highly restricted to activated T and NK cells [10].

In the absence of infection and during normal immune responses, CXCL13 and its receptor CXCR5 are involved in the homing of B-cells and follicular B-helper T cells into primary follicles in lymph nodes and spleen [11], and in germinal center formation and lymphoid organogenesis. Thus, CXCL13 and CXCR5-deficient mice exhibit impaired development of Peyer’s patches and lymph nodes due to the lack of organized follicles. Moreover, immunization with T-cell-dependent antigen in the context of CXCL13 knockout phenotype led to the formation of misplaced and abnormally small germinal centers in lymph nodes and spleens [12].

Generation of B cells with potential for autoantibody production is a common occurrence under normal physiological conditions. Such natural autoantibodies are, however, low affinity IgM antibodies that exhibit wide-spectrum reactivity and strong preference for soluble self antigens over those expressed on cell surface [13,14]. Autoreactive low-affinity B cells undergo apoptosis and, therefore, are unlikely to present any danger to a healthy organism.

In a chronically-inflamed environment, ectopic germinal centers form within affected (often non-lymphoid) tissues. CXCL13 over-expression by FDC in these germinal centers is accompanied by dysregulation of interactions among FDCs, B cells and follicular Th cells [15] and reduced elimination of autoreactive B cells. Subsequent, antigen-driven generation of affinity-matured long-lived plasma cells and memory B cells producing high affinity IgG autoantibodies contributes to the development of autoimmune and inflammatory disorders.

Th17 cells, a subset of the Th family, produce cytokines IL17, IL21 and IL22 and, under normal circumstances, play an important role in host defense against extracellular pathogens (e.g., bacteria, fungi). Under pathological conditions, however, Th17 cells have been linked to the development of autoimmune inflammation, allergic disorders and cancer [16-18]. In the context of CXCL13 biology, Th17 cells are of particular interest as both human and mouse Th17 cells express CXCR5 receptors and are able to migrate towards CXCL13 *in vitro* [8]. Human allo-reactive and pathogen-specific Th17, but not Th1 or Th2, clones were shown to express CXCL13, which may contribute to optimal Th17-B cell interactions necessary for antibody production [19,20]. Moreover, statistically significant correlation between IL-17 and CXCL13 levels in synovial fluid of patients with rheumatoid arthritis has been observed. Dramatic increases in myelin-specific Th17 cells in peripheral blood of patients with relapsing-remitting MS correlated with disease activity [21]. In patients with progressive MS, Th17 cells in cooperation with Tfh and activated B cell subsets have been shown to play a critical role in systemic inflammation associated with the development of meningeal ectopic lymphoid follicle-like structures (ELFs) and progression of the disease [22].

The family of autoimmune and inflammatory disorders where CXCL13 appears to be involved in disease pathogenesis and constitutes an attractive therapeutic target includes, among others, Multiple Sclerosis (MS) [23-26], Rheumatoid Arthritis (RA) [27-30]; Hashimoto’s thyroiditis [31], chronic gastritis/MALT lymphoma [32,33], graft rejection syndrome [34], Sjogren’s Syndrome [35]; Systemic Lupus Erythematosis [36], and Myastenia Gravis [37]. The mechanism of action for CXCL13-targeting treatments would involve blockade of CXCL13 interaction with its receptor resulting in inhibition of B, Tfh and Th17 cell migration and subsequent interference with the formation of ectopic germinal centers and development of tissue inflammation. Described below is the derivation and testing of a novel monoclonal anti-CXCL13 antibody that binds human, cynomolgus monkey and mouse CXCL13. We demonstrate that this monoclonal antibody is able to inhibit *in vitro* functional activity of human and mouse CXCL13 and present efficacy data of its murine analog in murine models of autoimmunity.

## Results

### Generation of human anti-CXCL13 antibody

**Figure 1:**
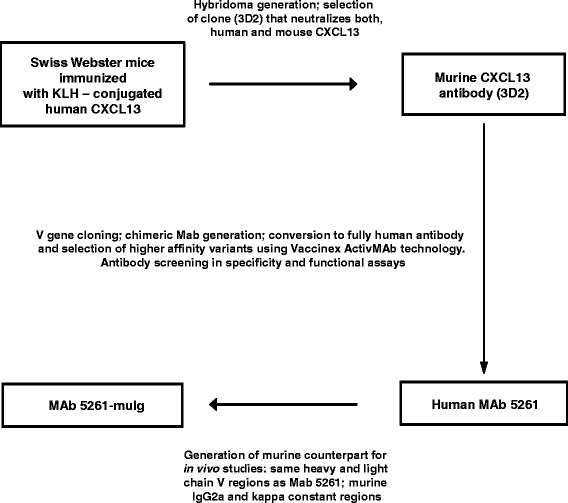
**Generation of MAb 5261 and its murine counterpart.**

Human anti-CXCL13 antibody, MAb 5261, was generated as described in detail in Methods section and illustrated in Figure 1. First, mouse hybridoma was produced by fusing myeloma cells with splenocytes from a mouse immunized with human CXCL13. Mouse monoclonal antibody, selected based on its ability to bind both human and mouse CXCL13, was then used as a source of V genes for the generation of the mouse-human antibody chimera. Humanization of the chimeric antibody and selection of higher affinity variants using Vaccinex’s proprietary ActivMAb® technology, led to the creation of anti-human CXCL13 antibody MAb 5261. For the *in vivo* experiments, we generated a MAb 5261-based chimeric antibody containing the human V genes and mouse constant domains (Figure 1).

To confirm CXCL13 specificity of MAb 5261 and MAb 5261-muIg we employed the following assays (data not shown): ELISA on a panel of recombinant human, murine and cynomolgus monkey CXCL13, human CXC chemokines most homologous to CXCL13 (CXCL12, CXCL8, CXCL10, and CXCL9; [38]) and various non-specific antigens (e.g., streptavidin, bovine serum albumin, human serum albumin, insulin, hemoglobin); flow cytometry on a panel of cell lines; and IHC on a panel of 31 normal human tissues. We found both antibodies to be specific for recombinant human, murine and cynomolgus monkey CXCL13. The binding affinity on CXCL13 from these species was determined by Biacore to be 5 nM for both human and chimeric antibodies. In addition, MAb 5261 efficiently bound CXCL13 from native sources (data not shown): human (from supernatants collected from IFN-γ-stimulated human monocytic cell line THP-1) and mouse (from CXCL13-rich organ extracts from TNF-α transgenic mice).

### Inhibition of CXCL13-mediated chemotaxis

**Figure 2:**
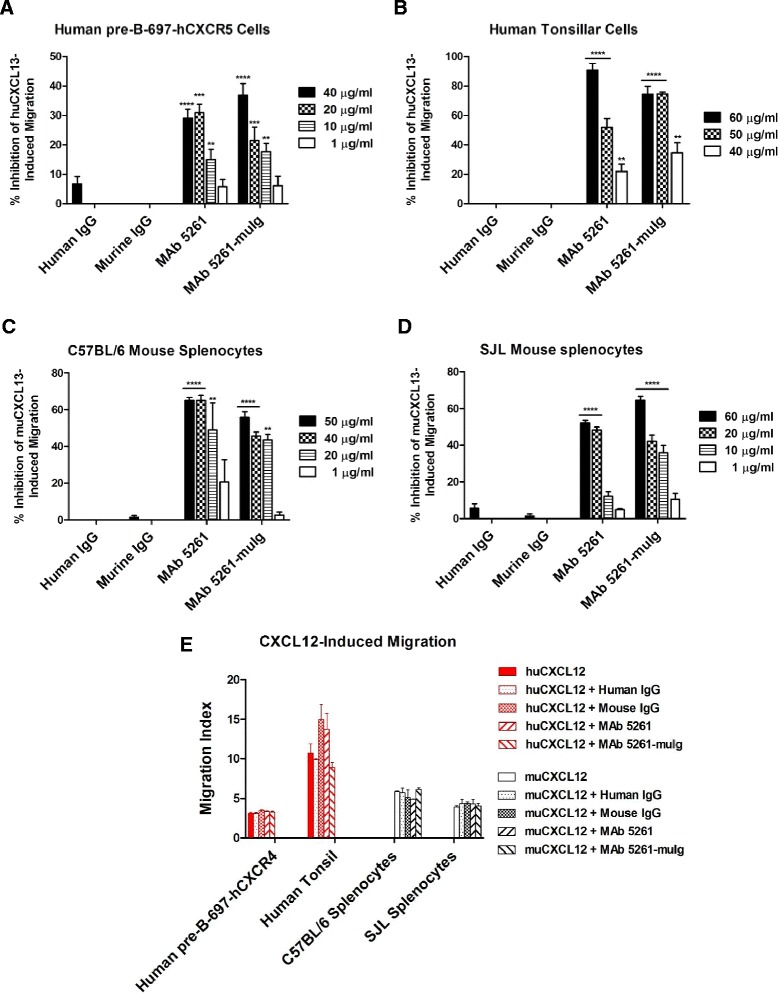
**Effect on CXCL13- (A-D) and CXCL12- (negative control; E) induced migration of human and murine cells.** Human pre-B-697-hCXCR5 cells **(A, E)**, human pre-B-697-hCXCR4 cells **(E)**, human tonsillar cells **(B, E)**, C57BL/6 splenocytes **(C, E)**, and SJL mouse splenocytes **(D, E)** were seeded into Transwell inserts. Diluted chemokines +/− antibodies were added into the lower chambers and plates were incubated for 2 h at 37°C (50 ug/ml of each antibody were used in CXCL12-induced migration assay). Inserts were removed and Alamar blue was added to the wells and incubated at 37°C overnight. Fluorescence was measured at wavelengths of 530 nm and 590 nm and the percent inhibition of chemokine-induced migration **(A-D)** or migration index **(E)** was calculated. Data represents an average of the measurements from at least three independent experiments + SEM. Statistical significance (relative to the isotype controls) was evaluated using one-way ANOVA followed by Bonferroni’s multiple comparison post test **** P < 0.0001; *** P < 0.0003; **P < 0.01.

The ability of MAb 5261 and MAb 5261-muIg to inhibit human CXCL13-induced cell migration was confirmed using stable cell line human pre-B-697-hCXCR5 and primary human tonsil cells (Figure 2A and B, respectively). The effect on mouse CXCL13-mediated chemotaxis was demonstrated using splenocytes from C57BL/6 and SJL/J mice (Figure 2C and D, respectively), where we observed a concentration-dependent inhibition of splenocyte migration towards CXCL13 by each MAb. Migration of human pre-B-697-hCXCR4 cells or mouse splenocytes towards human or murine CXCL12 respectively, was used to confirm specificity of each anti-CXCL13 MAb (Figure 2E). In summary, both MAb 5261 and MAb 5261-muIg met functional acceptance criteria that included inhibition of CXCL13-induced migration without affecting CXCL12-induced migration.

### Inhibition of human CXCL13-mediated endocytosis of human CXCR5 receptor

Characteristically of all chemokine receptors, binding of a chemokine leads to an internalization of the ligand-receptor complex with subsequent activation of the associated intracellular signaling cascade [39]. Burkle and colleagues [40] demonstrated that incubation of human CXCL13 with chronic lymphocytic leukemia (CLL) cells expressing high levels of functional CXCR5 receptor led to time- and dose-dependent receptor endocytosis. We adapted a flow cytometry-based method described in Burkle et al. to evaluate the ability of MAb 5261 and MAb 5261-muIg to inhibit CXCL13-mediated CXCR5 receptor internalization in human cells.

**Figure 3:**
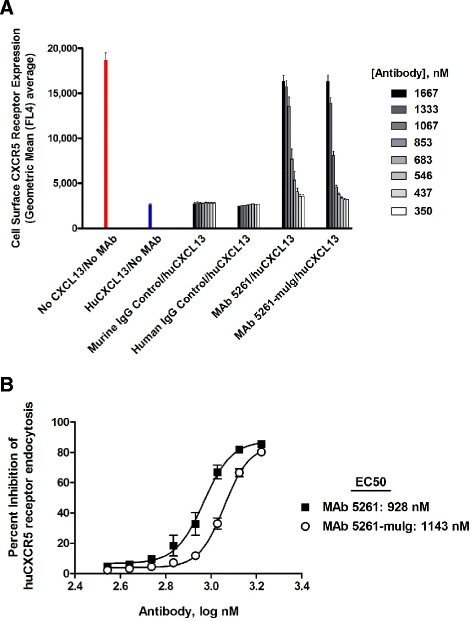
**Inhibition of human CXCL13-mediated internalization of human CXCR5 receptor.** Human pre-B-697-huCXCR5 cells were pre-blocked with anti-human Fc for 15 min at 37°C, incubated with a pre-complexed huCXCL13/antibody mix for 2 h at 37°C, and subsequently stained with anti-human CXCR5 antibody for flow cytometric analysis. **(A)** Cell surface CXCR5 receptor expression. Geometric mean values were determined by FlowJo 7.6 software. **(B)** EC50 values were calculated from sigmoidal dose response curves (shown on the graph) with R^2^ values equal to 0.9 (MAb 5261) and 0.98 (MAb 5261-muIg). Data points represent an average of measurements obtained from 3 independent experiments.

Figure 3 shows the effects of MAb 5261 and MAb 5261-muIg on human CXCR5 receptor expression on the surface of human pre-B-697-hCXCR5 cells treated with human CXCL13. Both antibodies efficiently and in a titratable manner interfered with CXCL13-induced internalization of the CXCR5 receptor.

### Murine anti-CXCL13 antibody affects splenic homing of adoptively transferred B cells in naïve BALB/c mice

**Figure 4:**
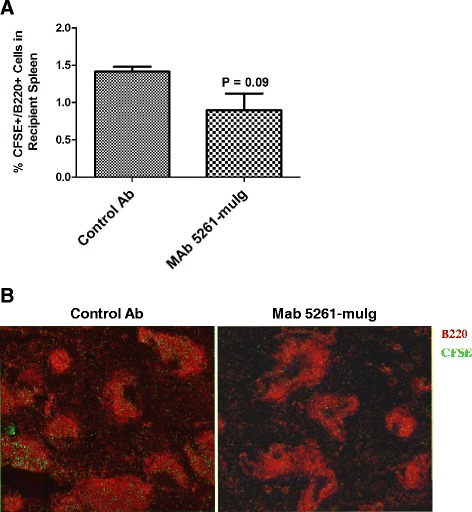
**MAb 5261-muIg-mediated inhibition of B cell migration into the spleens of naïve BALB/c mice.** Recipient mice were treated with 30 mg/kg of an Isotype Control Ab or MAb 5261-muIg intraperitoneally twice a week for 2 weeks before adoptive transfer of CFSE-labeled donor B cells (2x10^7^ cells per mouse; i.v.). **(A)** Percentage of CFSE+ B cells in mouse spleens (n = 3/group) as measured by flow cytometry. Flow data were analyzed using FlowJo 7.6 software. Statistical significance was evaluated using unpaired Student’s *t*-test **(B)** IHC on representative spleen sections where CFSE-labeled adoptively transferred cells are green and B cells are labeled red.

An *in vivo* functional assay was subsequently performed to determine if MAb 5261-muIg would inhibit the primary function of CXCL13, namely the homing of B cells to the follicles in secondary lymphoid organs. To this end, an adoptive transfer experiment was conducted in female BALB/c mice where B cells were isolated from spleens of untreated donor mice, labelled with CFSE, and injected intravenously into recipient mice that were pre-treated with bi-weekly i.p. injections of 30 mg/kg of either MAb 5261-muIg or control antibody for 2 weeks prior to adoptive transfer. Twenty four hours post-transfer, spleens were collected from the recipient mice and analyzed by flow cytometry and IHC to detect the presence of labeled B cells. As shown in Figure 4, migration of CFSE-labeled donor B cells into recipient spleens showed a trend to being reduced in MAb 5261-muIg-treated animals, indicating that this anti-CXCL13 specific antibody impedes B cell homing to a secondary lymphoid organ.

### Effect of murine anti-CXCL13 antibody on isotype class switching, frequency of high affinity NP-specific IgG1 producing cells and on the size and number of germinal centers in spleens of mice immunized with alum-precipitated NP-KLH

Arnold et al. reported an impaired germinal center response in mice with CXCR5-deficient T cells that manifested in a reduction in size and number of germinal centers with a reduction in the frequency of germinal center B cells and parallel reduction in frequency of NP-specific IgG1-secreting cells in spleens and bone marrow [41]. To determine if antibody-mediated inhibition of CXCL13 function would have a similar effect on formation of germinal centers in secondary lymphoid organs and isotype class switching by naïve B cells, we treated C57BL/6 mice immunized with alum-precipitated thymus-dependent antigen, 4-Hydroxy-3-nitrophenylacetyl conjugated keyhole limpet hemocyanin (NP-KLH) with MAb 5261-muIg or an appropriate isotype control antibody.

**Figure 5:**
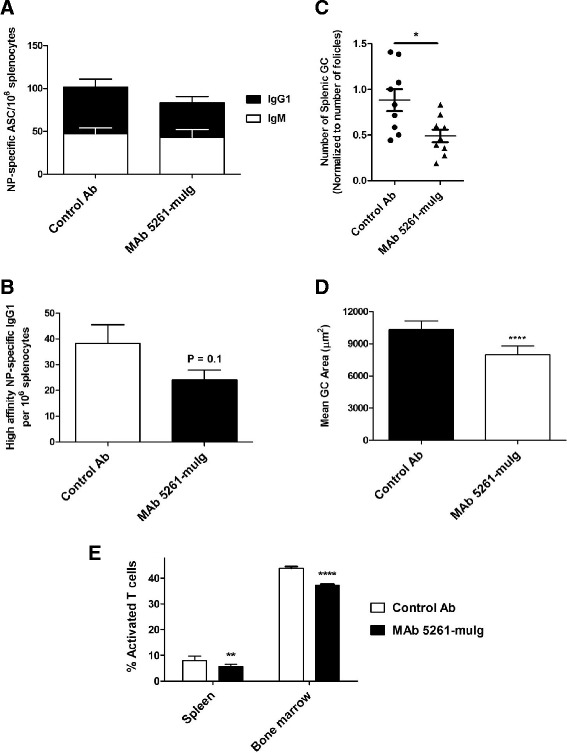
**Effect of murine anti-CXCL13 antibody on germinal center formation in C57BL/6 mice challenged with NP-KLH.** Mice were treated with 30 mg/kg of MAb 5261-muIg antibody and corresponding mouse isotype control, on days −3, 0 (the day of the immunization), 4 and 7 (n = 9/group). **(A)** Total IgM and IgG1 anti-NP antibody-secreting cells were detected by ELISPOT on wells coated with NP14-BSA. **(B)** The frequencies of high affinity IgG1 anti-NP-secreting cells were determined by ELISPOT on wells coated with NP4-BSA. **(C)** Number of germinal centers (n = 9 mice/group). Follicles were hand counted. Number of PNA+ germinal centers in relation to total number of follicles per group was calculated using ImagePro software. **(D)** Mean germinal center area. Area of each PNA+ germinal center for each group was calculated using ImagePro software by PNA+ pixel area and then converted to μm. The numbers of GC analyzed were: 201 in Control group and 102 in MAb 5261-muIg-treated group. **(E)** Percentages of activated T cells (CD4+/CD62Llow/CD44+) in spleens and bone marrow. Statistical significance was evaluated by Student’s unpaired *t*-test. *P = 0.0125; **P = 0.0016; ****P < 0.0001; P = 0.2 and 0.71 for IgG1 and IgM portions of the graph, respectively **(A)**.

Similar to the results described by Arnold and colleagues, on day 12 post-immunization, no difference in the frequency of splenic NP-specific IgM-secreting cells was observed between control and MAb 5261-muIg treated groups (Figure 5A). However, the mean frequency of class-switched NP-specific IgG1-secreting cells (both total (Figure 5A) and high-affinity (Figure 5B) antibody) was reduced in the animals treated with MAb 5261-muIg. The proportion of high affinity antibodies in the total IgG1 population (approximately 70% in each treatment group) was not affected, indicating a defect in affinity maturation had not occurred, but rather a reduction in the number of antibody-producing B cells in the germinal centers of treated animals. We also observed a significant reduction in number and size of the germinal centers in the spleens of animals treated with MAb 5261-muIg compared with the control group (Figure 5C and D, respectively), and a reduction in numbers of activated CD4 + T cells in spleens and bone marrow of MAb 5261-muIg antibody-treated animals (Figure 5E). These data suggest that the potential mechanism of action of our anti-CXCL13 antibody involves inhibition of CXL13-mediated recruitment of B and CXCR5+ CD4+ T cells into lymphoid follicles with subsequent interference with the formation and expansion of germinal centers.

### Murine anti-CXCL13 antibody reduces the severity of Collagen-Induced Arthritis

Collagen-induced arthritis in mice and rats is a well-established model of human rheumatoid arthritis [42]. The disease is typically induced by intradermal injection of bovine type II collagen emulsified in Complete Freund’s Adjuvant (CFA) and is characterized by the production of mouse collagen antibodies and subsequent progressive development of arthritis in the paws. The role of CXCL13 in CIA pathogenesis has been previously highlighted. For example, Zheng et al. demonstrated that treatment with polyclonal anti-CXCL13 antibodies reduced disease severity and inhibited formation of germinal centers in the joints of mice affected by CIA [43]. Similarly Finch and colleagues reported that in mice with an established CIA, CXCL13 neutralization with rat anti-murine CXCL13 antibodies led to B cell depletion in the lymphoid tissues and significantly reduced severity of joint destruction [44].

To assess the therapeutic potential of MAb 5261-muIg in CIA, arthritis was induced in male DBA/1 J mice. The mice were subjected to either prophylactic or therapeutic treatment regimens that consisted of bi-weekly injections of 30 mg/kg of antibody. Anti-CXCL13 antibodies were delivered intraperitoneally, while positive control antibody, Etanercept (Enbrel®, Amgen), was delivered subcutaneously. Prophylactic treatment began on day 20 post-collagen immunization, 1 day prior to the booster immunization and continued for 3 weeks. Therapeutic treatment was initiated when the group mean arthritic index (AI) reached 3–4 (approximately on day 30 post-initial immunization) and continued for 2 weeks.

**Figure 6:**
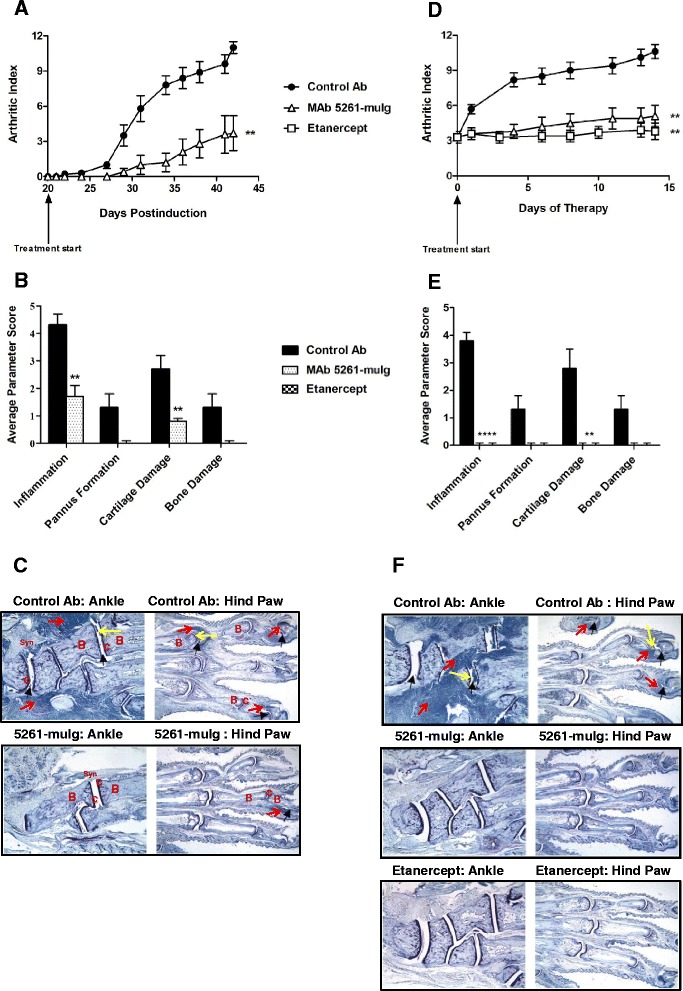
**Effect of prophylactic and therapeutic treatment with MAb 5261-muIg on CIA progression. (A-C)** Prophylactic treatment. **(D-F)** Therapeutic treatment. **(A and D)** Arthritic indexes (AI). **(B and E)** Summary of the histological analysis. Statistical significance was evaluated using unpaired Student’s *t-*test (n = 10 mice/group); **P < 0.001; ****P < 0.0001. **(C and F)** Toluidine blue staining of the selected limbs. **(C)**: AI at termination: 11 (control), 4 (MAb 5261-muIg). **(F)**: AI at termination: 10 (control), 6 (MAb 5261-muIg), 4 (Etanercept). B – Bone, C – Cartilage, Syn – Synovial tissue. Thick arrows identify affected joints; yellow arrow – pannus; red arrow – infiltration of inflammatory cells. Results shown are representative of at least three independent experiments.

As evident from Figure 6A-C, prophylactic treatment with MAb 5261-muIg resulted in a delay in disease onset and reduced rate of disease development. Therapeutic treatment with MAb 5261-muIg (Figure 6D-F), although initiated after onset of arthritis, resulted in a significantly curtailed disease development, similar to positive control Etanercept. In both study designs, joints of the control mice exhibited severe inflammation, moderate cartilage damage with minimal pannus formation and bone resorption in the ankle and all digit joints (Figure 6C and F). Minimal inflammation and cartilage damage were observed in the mice prophylactically treated with MAb 5261-muIg (Figure 6C). In the therapeutic cohort, no lesions were observed in hind paws and ankles of animals treated with either MAb 5261-muIg or Etanercept (Figure 6F). Despite the effect on disease development and progression, serum anti-collagen II antibody titers were not affected by the treatment with MAb 5261-muIg (data not shown).

### Murine anti-CXCL13 antibody attenuates symptoms in experimental autoimmune encephalomyelitis

Experimental Autoimmune Encephalomyelitis, an animal model of multiple sclerosis, is a demyelinating disease of the CNS that progressively results in escalating degrees of ascending paralysis with inflammation primarily targeting the spinal cord. EAE can assume an acute, chronic or relapsing-remitting course and can be induced in mice either actively by immunization with myelin antigens (e.g., myelin oligodendrocyte glycoprotein (MOG); proteolipid protein (PLP)) or passively via adoptive transfer of myelin-specific CD4+ T cells from actively immunized donors to naïve recipients [45,46].

Exploring the role of CXCL13 in EAE pathogenesis, Bagaeva et al. demonstrated that follicles containing B-cells and CXCL13-expressing dendritic cells formed in inflamed meninges of mice with relapsing-remitting EAE with levels of CXCL13 expression rising steadily throughout the course of disease [47]. Moreover, CXCL13-deficient mice experienced mild disease with decreased relapse rate, while blockade of CXCL13 with anti-CXCL13 MAb led to disease attenuation in passively induced EAE in B10.PL mice.

**Figure 7:**
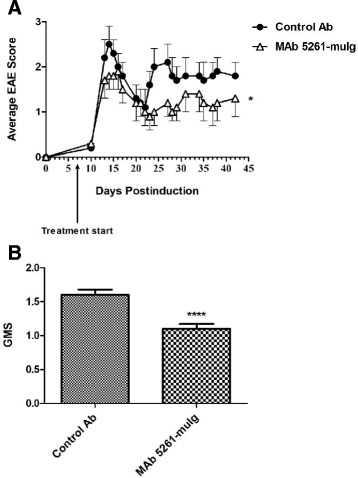
**Effect of MAb 5261-muIg on relapsing-remitting EAE in SJL/J mice.** Mice (n = 15 per group) were treated starting on day 7 post-PLP_139–151_/CFA immunization with 30 mg/kg of MAb 5261-muIg or an isotype control antibody i.p., once a week for 8 weeks. **(A)** Average EAE scores during the course of the disease. **(B)** Group mean scores (GMS). Statistical significance was evaluated using unpaired Student’s *t-*test. *P < 0.1; ****P < 0.0001.

We initially tested mouse antibody 3D2, the prototype of MAb 5261 (Figure 1), in SJL/J relapsing-remitting model of EAE where this MAb attenuated disease progression in 3 independent experiments (data not shown). Encouraged by the results, we used the same model of EAE to evaluate the effects of MAb 5261-muIg. The treatment started on day 7 post-immunization and consisted of weekly intraperitoneal injections with 30 mg/kg of the antibodies. As evident from data shown in Figure 7, the treatment with MAb 5261-muIg resulted in significant reduction in the severity of relapsing-remitting EAE.

To separate EAE-inducing T cell subsets and, thereby, gain better understanding of the mechanism of action of MAb 5261-muIg in EAE models, we employed a passive method of EAE induction. For that, we chose two CD4+ cell subsets: IL-17-secreting Th17 cells and IFN-γ-secreting Th1 cells. As previously reported by Jager et al., in adoptive transfer model of EAE both cell types produced typical demyelinating lesions in white matter, with twice as many lesions present in Th17-induced animals [48]. Th1 cell recipients developed classic paralytic symptoms of EAE, while the recipients of Th17 cells in addition to the classical symptoms, also developed atypical signs of the disease that included ataxia, severe imbalance, weight loss and high mortality rate [49,50]. Peters and colleagues demonstrated that in a mouse model of passive EAE, adoptively transferred CXCR5+ Th17 cells, but not other Th cells subsets (Th1, Th2 or Th9), induced development of ectopic lymphoid follicle-like structures (ELFs) in the CNS through the upregulation of CXCL13 expression and action of Th17-specific cytokine IL-17 [51]. Based on the latter observation, we hypothesized that MAb 5261-muIg would predominantly affect Th17-mediated disease.

**Figure 8:**
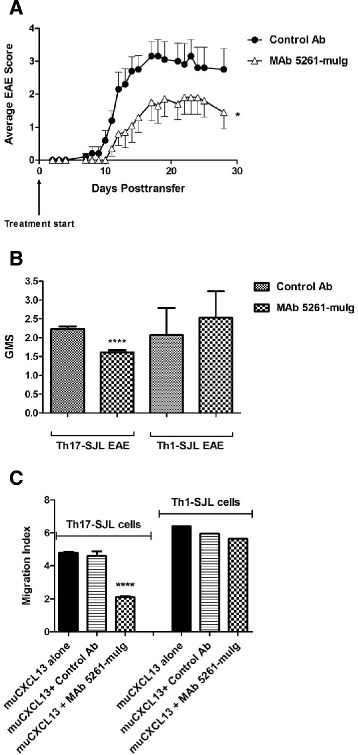
**Effect of MAb 5261-muIg on adoptively transferred EAE in SJL/J mice.** Mice were treated i.p. with 30 mg/kg of antibody starting on the day of the adoptive transfer and twice a week thereafter for the total of 4 weeks (n = 10 per group). **(A)** MAb 5261-muIg in Th17-SJL EAE. **(B)** MAb 5261-muIg in Th17 vs. Th1 SJL-EAE: Th17 EAE: GMS +/− SE (GMS calculated from three independent experiments); Th1 EAE: GMS +/− SD (Results of one experiment +/− SD). **(C)** MAb 5261-muIg effect on muCXCL13-induced migration of Th17 and Th1 cells from successful SJL/J passive EAE transfer experiments (concentrations: 5 μg/ml muCXCL13; 50 μg/ml of each antibody). Results are presented as average of triplicate measurements +/− SE (except for Th1 cell migration data which originated from a single measurement). Statistical significance was evaluated using unpaired Student’s *t-*test **(A)** and one-way ANOVA followed by Bonferroni’s multiple comparison post test (**B** and **C**). **** P < 0.0001; *P < 0.1.

Passive EAE was induced in female SJL/J mice by adoptive transfer of Th1 or Th17 enriched cells differentiated from T cells of donors actively immunized with PLP_139–151_. Starting on the day of the adoptive transfer, the animals were treated with 30 mg/kg of the antibodies, intraperitoneally, twice a week for a total of 4 weeks. The mice were scored for the clinical signs of the disease as described in Methods. MAb 5261-muIg significantly reduced the severity of Th17-induced EAE (Figure 8A and B), but failed to alleviate the symptoms of Th1-induced EAE (Figure 8B), indicating a specific mechanism of disease inhibition that involves Th17-mediated pathways. To further confirm the effect of MAb 5261-muIg on Th17 cells, we tested this MAb in a Th17-targeted chemotaxis assay where the antibody inhibited CXCL13-mediated migration of Th17 cells, without affecting migration of Th1 cells (Figure 8C).

### Tolerability of MAb 5261-muIg and MAb 5261 treatment

In both models, CIA and EAE, we found MAb 5261-muIg to be well tolerated, as no anti-drug antibody response (ADA) was detected by ELISA. Treatment regimen of bi-weekly intraperitoneal injections with 0.6 mg/ml MAb 5261-muIg typically resulted in steady serum levels of approximately 500 μg/ml of the antibody at study termination (usually at 2 weeks following the last antibody injection).

To evaluate the safety of our human anti-CXCL13 antibody, MAb 5261, we conducted a single-dose toxicology study at Ethox International, STS Life Sciences. Sprague Dawley rats were injected intravenously with MAb 5261 in doses ranging from 0.01 to 100 mg/kg. The antibody half-life was dose-dependent with values ranged from 1 to 14 days. No toxicity or immunogenicity was observed in any of the treatment groups.

## Discussion

CXCL13 is a homeostatic chemokine that is constitutively expressed in secondary lymphoid tissue and involved in lymphoid organogenesis. In the setting of disease, however, the levels of CXCL13 have been shown to significantly increase. In fact, in a number of the autoimmune disorders (e.g., MS [52], SLE [53], Sjogren’s syndrome [38] and RA [27,54]), CXCL13 is considered a biomarker indicative of disease severity.

Antibody-based therapeutics targeting chemokine–mediated pathways are promising due to the specificity of their effect and the ability to enhance immune responses through activation of complement-dependent or antibody-dependent cytotoxicity (CDC and ADCC, respectively) that might be useful in certain disease states. In recent years, several anti-chemokine antibody therapeutics have emerged and been tested in clinical trials [55]. One of them, fully human anti-CXCL10 antibody MDX-1100 (aka BMS-936557; Bristol Myers Squibb) has demonstrated efficacy in Phase II clinical trials involving patients with rheumatoid arthritis [56] and ulcerative colitis [57].

We describe here a novel human anti-human CXCL13 antibody, MAb 5261, whose murine analog, MAb 5261-muIg, has demonstrated efficacy in two well-characterized mouse models of autoimmunity: CIA (both prophylactic and therapeutic models) and passively and actively induced models of relapsing-remitting EAE.

In the model of an established arthritis, the effect of antibody 5261 was comparable to that of Etanercept, a drug routinely prescribed to treat moderate to severe RA. Etanercept is a soluble TNF-α receptor fusion protein. Although effective in RA, Etanercept, as other TNF-α blockers, is an immunosuppressant and is associated with a number of serious side effects (unusual cancers, infections, congestive heart failure, CNS problems, and autoimmunity) [58], thus suggesting a need for safer treatment options, one of which might be an antibody targeting CXCL13.

In the EAE model, MAb 5261-muIg reduced severity of the disease mediated by CXCR5+ Th17 cells presumably through inhibition of cell migration into the CNS. While it has been widely accepted that CNS inflammation in MS patients is mediated mainly by the IFN-γ-secreting Th1 cells, in recent years, Th17 cells have emerged as major players in MS due to their high pathogenic potential through the production of proinflammatory cytokine IL-17A. In addition, as compared to Th1 cells, Th17 cells have a higher proliferative capacity, higher expression of activation markers (e.g., CD5, CD69, human leukocyte antigen-DR) and adhesion molecules (e.g., CD49d, CD6 and MCAM (CD146)) required for T cell penetration into the brain, and reduced susceptibility to Treg-induced suppression [59]. Clinically, in patients with relapsing-remitting MS, the frequency of Th17 cells is significantly elevated in cerebrospinal fluid (CSF) as compared to blood; the frequency of Th17 cells in CSF is significantly higher during relapse than in remission, while the frequency of Th1 cells remains relatively stable [59]. Moreover, in patients with aggressive MS that were treated with ablative chemotherapy and autologous hematopoietic stem cell transplantation, the absence of new relapses and inflammatory brain lesions 2 years following treatment was linked to diminished Th17 and Th1/17 cells (newly defined subset of Th cells that express cytokines and transcription factors of both, Th17 and Th1 lineages), but not Th1 responses, implicating the former T-cell subsets’ involvement in MS pathogenesis [60].

Targeting Th17 cells, therefore, appears to be a reasonable strategy for developing next-generation treatments for MS. In fact, some of the approved MS therapies, including Glatiramer Acetate [61,62], FTY720 [63] and IFN-β [64], are capable of reducing Th17 responses in both experimental autoimmune encephalomyelitis models [61,62] and in patients with multiple sclerosis [63,64]. On the other hand, ustekinumab, an IL-12/IL-23 monoclonal antibody targeting Th17 differentiation, although well-tolerated, failed to demonstrate efficacy in a Phase II clinical study involving patients with relapsing-remitting MS [65]. A monoclonal antibody targeting Th17 trafficking into the CNS, such as the anti-CXCL13 antibody described herein, may serve as a more effective strategy to block Th17 cell-mediated pathologic effects and thereby alleviate disease symptoms associated with MS.

As shown in Figure 4, MAb 5261-muIg significantly inhibited B cell migration into spleens of naïve Balb/c mice, while Kramer and colleagues recently demonstrated that CXCL13 neutralization with MAb 5261-muIg in a NOD mouse model of Sjogren’s syndrome caused reduction in salivary gland inflammation by inhibiting B cell recruitment into submandibular gland tissue [38]. In an attempt to decipher the mechanism of action of anti-CXCL13 antibody in animal models, Finch et al. [66] examined the integrity of splenic architecture in BALB/c mice immunized with alum-precipitated DNP-OVA and treated with rat anti-mouse CXCL13 antibody MAb 470 (R&D Systems, clone 143614). They found that anti-CXCL13 treatment caused a disruption in splenic follicular structure accompanied by a significant reduction in germinal center frequency. The serum titers of DNP-OVA-specific IgG and the frequency of the germinal center B cells were not affected by the treatment. Similarly, MAb 5261-muIg caused a decline in the number and size of splenic germinal centers of the C57BL/6 mice immunized with alum-precipitated NP-KLH (Figure 5C and D), but not a reduction in the frequency of B220+/PNAhigh/CD38low GC B cells (data not shown). While we have not assessed the serum titers of NP-specific IgG, we did observe a significant decrease in the frequency of NP-specific IgG1-secreting cells, consistent with the effects reported by Arnold et al. [40] in mice with CXCR5-deficient T cells.

## Conclusions

In this report we describe the derivation and functional testing of a novel human anti-human CXCL13 antibody, MAb 5261, and provide its detailed pharmacological characteristics. The antibody binds to human, mouse and cyno CXCL13 with an affinity of approximately 5 nM. It efficiently and specifically inhibits CXCL13-induced chemotaxis of human and murine B cells as well as huCXCL13-mediated CXCR5 receptor endocytosis. Importantly, the antibody is non-toxic as revealed by preclinical single-dose rat PK study.

Its murine counterpart, MAb 5261-muIg, created specifically for *in vivo* preclinical studies, effectively inhibits B and activated T-cell recruitment into secondary lymphoid organs and germinal center formation in mice immunized with thymus-dependent antigen and autoimmune mice. Through its effect on the migration of immune cells, MAb 5261-muIg inhibited progression and severity of relapsing-remitting EAE and, specifically, Th17-induced EAE in SJL mice and alleviated symptoms of arthritis in mice immunized with Type II collagen.

Our results, therefore, indicate that CXCL13 neutralizing MAb 5261 antibody has potential for treatment of autoimmune disorders, including Multiple Sclerosis and Rheumatoid Arthritis

## Methods

### Materials

Chemokines/Cytokines: recombinant human CXCL13 (Peprotech; 300–47); recombinant murine CXCL13 (Peprotech; 250–24); recombinant murine CXCL12 (Peprotech; 250-20A); recombinant human CXCL12 (Peprotech; 300-28A); recombinant murine IL-12 (R&D Systems; 419-ML/CF); recombinant murine IL-2 (R&D Systems; 402-ML); recombinant murine IL-6 (R&D Systems; 406-ML/CF); recombinant human TGF-β (R&D Systems; 240-B); recombinant murine IL-23 (R&D Systems; 1887-ML).

Antibodies: rat anti-mouse MAb 470 (R&D Systems; MAB470); mouse anti-human MAb 801 (R&D Systems; MAB801); goat anti-human AF801 (R&D Systems; AF801); goat anti-mouse AF470 (R&D Systems; AF470); anti-human CXCR5-Alexa 647 (BD Pharmingen; 558113); anti-human CXCR4-APC (R&D Systems; FAB170A); donkey anti-mouse IgG-HRP (Jackson ImmunoResearch; 715-035-150); donkey anti-rat IgG-HRP (Jackson ImmunoResearch; 712-035-150); B220-FITC (BD Biosciences; 553087); CD21/35-APC (BD Biosciences; 558658); CD23-PE (BD Biosciences; 553139); anti-human CD32 (Biolegend; 303202); anti-murine IL-4 (clone 11B11; BioXcell, BE0055); anti-murine CD3e (clone 145-2C11; BioXcell; BE0001-1); anti-murine CD28 (clone PV-1; BioXcell; BE0015-5); anti-murine IFN-γ (clone XMG1.2; BioXcell; BE0055).

Reagents: PLP_139–151_ (Anaspec; 29213); MOG_35–55_ (Biosynthesis Inc.; 12668–01); NP-KLH (Biosearch Technologies; N-5060); Imject Alum (Thermo Fisher; PI-77161); NP-BSA (Biosearch Technologies; N-5050); Imject BSA (ThermoScientific; 77110); Imject KLH (ThermoScientific; 77600).

### Generation of anti-human CXCL13 antibody, MAb 5261

We initially produced hybridomas secreting mouse anti-human CXCL13 antibodies. Briefly, Swiss Webster mice were immunized with KLH-conjugated human CXCL13. After three immunizations, the spleen was harvested from the mouse with the highest anti-CXCL13 titer and hybridomas were generated by fusion of spleen cells with SP2/0 myeloma cells using standard procedures. Hybridoma cells were originally grown in DMEM supplemented with 10% FBS, 4.5 g/ml glucose, L-glutamine and Na pyruvate. The cells were subcloned by limiting dilution. Twenty clones were expanded and gradually adjusted to serum-free growing conditions in Excell 610-HSF hybridoma serum-free medium. Hybridoma clones were screened by ELISA for binding to human and murine CXCL13. The antibody, called 3D2, that demonstrated dual specificity on both murine and human CXCL13 became a prototype for human anti-human CXCL13 generation.

The V genes were isolated from 3D2 hybridoma using standard methods. The VH gene was cloned into a mammalian expression vector that contained the human gamma 1 heavy chain gene, creating a full-length chimeric heavy chain. The VK was cloned into a mammalian expression vector that contained the human Kappa constant gene, creating a full-length chimeric light chain. In order to make the chimeric antibody, the expression vectors harboring the chimeric heavy chain and the chimeric light chain were co-transfected into CHO-S cells. The MAb was secreted by the cells and harvested following a 3–6 day expression period. The resulting MAb was purified using Protein A chromatography, and characterized. The resulting chimeric IgG1 antibody was specific for human and murine CXCL13 by ELISA, and demonstrated similar affinity on murine and human CXCL13 and similar functional activity as parental mouse antibody.

Generation of human anti-CXCL13 antibody through humanization of chimeric antibody was accomplished as follows. The following modifications were introduced to FWR regions of heavy and light chains from the chimeric antibody: (i) a putative N-linked glycosylation site (NLT) in the heavy chain was replaced with “SLT”; and (ii) a single serine to methionine mutation was introduced at position 31 in light chain CDR1. The modifications resulted in generation of human IgG1 antibody, MAb 5261, which demonstrated significant improvement in affinity as compared to the parental mouse anti-CXCL13 antibody.

### Generation of murine counterpart of MAb 5261 for use in animal studies

MAb 5261 contains human heavy and light variable and human IgGamma1-F allotype as well as human kappa. Its murine counterpart, MAb 5261-muIg (a.k.a MAb 5378, [38]), had been engineered as a mouse IgG2a isotype. The murine antibody contains the same human heavy and light chain variable genes as MAb 5261 along with mouse IgGamma2a constant region and mouse kappa. Vaccinex has engineered common restriction sites among all of our heavy and light chain expression plasmids such that changing isotypes and species can be achieved through restriction digestion, ligation, and transformation. Specifically, for the MAb 5261 heavy chain, the variable region of the gene was digested with restriction endonucleases *Bss*HII and *Bst*EII and ligated into comparable sites in an expression plasmid that contains the mouse IgGamma2a constant region in order to make the heavy chain for MAb 5261-muIg. Similarly, for the MAb 5261 light chain, the variable region of the gene was digested with restriction endonucleases *Apa*LI and *Xho*I and ligated into comparable sites in an expression plasmid that contained the mouse IgKappa constant region in order to make the light chain for MAb 5261-muIg.

### Screening hybridoma supernatants for anti-CXCL13 antibodies by ELISA

Supernatants were collected and screened for binding to human and murine CXCL13 using the following protocol: plates were coated with 1 μg/ml of human or murine CXCL13 at room temperature overnight. After the plates were washed and blocked, antibodies (MAb 470 and MAb 801 used as standard) at 1 μg/ml - 0.02 μg/ml serial dilutions or hybridoma sups at 1:10–1:50,000 serial dilutions were added and incubated at room temperature for one hour. Plates were washed and secondary antibodies (donkey anti-rat IgG- HRP for MAb 470; donkey anti-mouse IgG-HRP for MAb 801 and hybridoma supernatants) were added at 1:20,000 dilution. Plates were incubated for 1 h at room temperature, washed, developed for 15 min in the dark and read at 450/570 nm.

### Affinity measurements by Biacore

Chemokines were immobilized on C1 chip in Acetate buffer (pH = 5): human CXCL13 at 1 μg/ml, murine CXCL13 at 0.3 μg/ml and negative control human CXCL12 at 0.5 μg/ml. Monoclonal anti-CXCL13 antibodies were diluted by 2-fold from 50 nM to 0.78 nM for binding to human CXCL13 and from 19 nM to 0.594 nM for binding to murine CXCL13. The curves were analyzed using Bivalent model.

### Generation of human pre-B-697-huCXCR5 and huCXCR4 stable cell lines

Packaging cell lines were generated by transfecting PA317 cells with LNCX2-hCXCR5 (or hCXCR4) retroviral constructs using “Fugene HD” transfection reagent (Promega). Human pre-B-697 cells were infected with retroviral sups collected of PA317-huCXCR5 or huCXCR4 cells using “spin infection” protocol: cells were centrifuged with retroviral sups at 2500 rpm for three hours at room temperature; the sups were removed and cells were allowed to recover in drug-free culture medium for twenty four hours. After that, the cells were placed under selection with 0.4 mg/ml G418. Once resistant cells reached ≥ 90% viability, they were flow sorted using either anti-huCXCR5 or anti-huCXCR4 antibodies into 96-well plates. Clones were expanded and screened by flow cytometry to select highest receptor expressors. Selected clones were then tested in migration assays to determine their functionality. If necessary, the clones were subcloned by limiting dilution one more time.

### Chemotaxis assay using human pre-B-697-huCXCR5 and huCXCR4 cells

Transwell tissue culture treated plates with 8 μm pore size and diameter of 6.5 mm (Corning Costar; 3422) were used. Human pre-B-697-huCXCR5 cells were used for huCXCL13-induced migration; human pre-B-697-huCXCR4 cells for, negative control, huCXCL12-induced migration. Cells were resuspended in chemotaxis medium (RPMI 1640 with L-glutamine, 10 mM HEPES, Penicillin-Streptomycin and 0.5% BSA) at 5×10^6^/ml and re-sensitized for thirty minutes at 37°C. Diluted chemokine (1 μg/ml huCXCL13; 0.1 μg/ml huCXCL12 in chemotaxis buffer) +/− antibodies are added into the lower chamber at 590 μl/well; pre-incubated for 15 min at room temperature. The cells were added at 100 μl (5×10^5^ cells) per upper chamber. Plates were incubated overnight at 37°C. Inserts were removed. Alamar blue was added at 60 μl per well; plates were incubated at 37°C for 4 h. Fluorescence was measured at wavelengths of 530 nm and 590 nm. Migration index and percent migration inhibition were calculated as follows:

$$ \%\ \mathrm{M}\mathrm{igration}\ \mathrm{index}=\frac{\mathrm{Specific}\ \mathrm{migration}\ \left(\mathrm{Chemokine} + \mathrm{M}\mathrm{A}\mathrm{b}\ \mathrm{or}\ \mathrm{Isotype}\ \mathrm{Control}\right)}{\mathrm{Spontaneous}\ \mathrm{M}\mathrm{igration}\ \left(\mathrm{No}\ \mathrm{chemokine}/\mathrm{No}\ \mathrm{antibody}\right)} $$

$$ \%\ \mathrm{M}\mathrm{igration}\ \mathrm{inhibition}=\frac{\left(\mathrm{Migration}\ \mathrm{Index}\ \left(\mathrm{Chemokine}\right)\hbox{-} \mathrm{M}\mathrm{igration}\ \mathrm{Index}\ \left(\mathrm{Chemokine} + \mathrm{M}\mathrm{A}\mathrm{b}\hbox{'}\right)\right)\ast 100}{\mathrm{Migration}\ \mathrm{Index}\ \left(\mathrm{Chemokine}\right)} $$

### Chemotaxis assay using murine splenocytes and human tonsil cells

Transwell tissue culture treated plates with 5 μm pore size and diameter of 6.5 mm (Corning Costar; 3421) were used. Mouse spleens and human tonsils were crashed with 5-ml syringe plunger; passed through 40-μm (spleens) or 70-μm (tonsils) filter. Red blood cells were lysed with RBC lysis buffer (eBioscience) for 5 min at room temperature. The cells were filtered and washed twice with the chemotaxis medium. The cells were resuspended at a density of 1×10^7^ cells/ml in chemotaxis medium and resensitized for 2–4 h at 37°C. Diluted chemokine (5 μg/ml of murine or human CXCL13 or 0.1 μg/ml of murine or human CXCL12) +/− antibodies were added into the lower chamber at 590 μl/well and pre-incubated for 15 min at room temperature. The cells were added at 100 μl (10^6^) cells per upper chamber. Plates were incubated for 2 h at 37°C. Inserts were removed. Alamar blue was added at 60 μl per well and incubated at 37°C overnight. Fluorescence was measured at wavelengths of 530 nm and 590 nm. Migration index and migration inhibition were calculated as described above.

### Human CXCR5 receptor endocytosis assay

Working stocks of antibodies were serially diluted from 100 μg/ml to 4 μg/ml. Human CXCL13 was diluted to 20 μg/ml in cold diluent buffer (RPMI + 0.5% BSA). Thirty microliters each of hCXCL13 and antibodies were combined and incubated overnight at 4°C. The next day, cells were resuspended in diluent buffer at 10^7^ cells/ml. Cells were pre-blocked with 10 μg/ml anti-human Fc block for 15 min at 37°C. Cells were incubated with huCXCL13/antibody mix (50 μl cells: 50 μl mix) for 2 h at 37°C and stained with 15 μl anti-human CXCR5 antibody (30 min at 4°C). Inhibition of endocytosis was calculated as follows: % Inhibition = 100 - [100*(0 CXCL13 - geomean)/(0 CXCL13 - 0 mAB)]

### Murine active immunization - induced experimental autoimmune encephalomyelitis

Relapsing-remitting (RR) disease was induced in 8–10 week-old female SJL/J mice (Charles River Laboratories) by subcutaneous immunization with PLP_139–151_ in 1 mg/ml Complete Freund’s Adjuvant (CFA) enhanced with 5 mg *Mycobacterium tuberculosis* strain H37RA (100 μl of PLP/CFA emulsion were injected into four sites on the back of the animal; 25 μl per site), using a pertussis toxin-free kit from Hooke Laboratories, Inc (EK-0120). The treatment started on day 7 post-immunization, lasted for 8 weeks and consisted of weekly i.p. injections of 30 mg/kg of anti-CXCL13 or control antibody. The clinical signs of the disease were evaluated at least 5 times a week using the following scoring system (adapted with modifications from Bagaeva et al. [47]: 0, normal behavior; 1, distal limp tail; 1.5, complete limp tail; 2, impaired righting ability; 3, ataxia; 4, paralysis of one hind leg; 4.5, paralysis of both hind legs; 5, full paralysis; and 6, death.

### Murine passively induced experimental autoimmune encephalomyelitis (adoptive transfer model)

The disease was induced in female 10–11 week-old SJL/J (Charles River Laboratories) mice by adoptive transfer of either Th1 or Th17 cells from immunized donors. First, syngeneic donors (7–8 week-old SJL/J mice) were immunized with PLP_139–151_/CFA emulsion as described above. On day 10 post-immunization, the animals were sacrificed and T cells were isolated from spleens and lymph nodes using the kit from Miltenyi Biotech (130-095-248). For T helper subset differentiation, the cells were plated with the irradiated APC (3400 rad) in media (RPMI1640/10% FBS/Na Pyruvate/Non-essential amino acids/2-mercaptoethanol/Penicillin-Streptomycin) containing 20 μg/ml PLP_139–151_ and either 20 μg/ml anti-muIL-4 and 10 ng/ml muIL-12 (**Th1** cells) or 30 ng/ml muIL-6, 3 ng/ml huTGF-β, 20 μg/ml anti-muIFN-γ and 20 μg/ml anti-muIL-4 (**Th17** cells). The cells were incubated at 37°C for 3 days. The cells were then mixed with freshly irradiated APC and resuspended in media containing 20 μg/ml PLP_139–151_ and either 20 U/ml muIL-2 (**Th1** cells) or 10 ng/ml muIL-23 (**Th17** cells). The cells were incubated at 37°C for 4 days after which the cells were collected, washed and cultured in media containing 2 μg/ml of each anti-muCD3e and anti-muCD28 antibody for 3 days. For the adoptive transfer, the mice were injected i.p. with 2×10^6^ cells in 100 μl PBS. Starting on the day of the adoptive transfer, the animals were given bi-weekly i.p. injections of 30 mg/kg of the antibodies for the total of 4 weeks.

### Adoptive transfer of B cells

B cells were isolated from untreated 8–12 week-old female BALB/c donor mice (Taconic Farms) using a kit (Miltenyi Biotech (130-090-862)). Donor B cells were labeled with 1 μM CFSE (Molecular Probes; C34554) and injected (2X10^7^ cells/i.v.) into syngeneic recipients pre-treated with i.p. injections of 30 mg/kg of either isotype control antibody or anti-CXCL13 antibody for 2 weeks pre-transfer. Twenty four hours post-transfer, recipients were sacrificed, spleens collected and analyzed by flow cytometry and IHC.

### Germinal center study

Male 8–10 week-old C57BL/6 mice (Jackson Laboratories) were immunized i.p. with 100 μg NP-KLH precipitated in 100 μl Imject Alum in 100 μl PBS. The animals were treated with i.p. injections of 30 mg/kg of anti-CXCL13 antibody or corresponding isotype control antibody on days −3, 0, 4 and 7 relative to the immunization day. The animals were euthanized on day 12 post-immunization. Spleens and bone marrow were collected from all animals. The spleens were bisected longitudinally for separate IHC and ELISPOT analyses. Bone marrow was used for FACS analysis.

Animal studies described above were conducted at the AAALAC (Association for Assessment and Accreditation of Laboratory Animal Care International) accredited animal husbandry facilities at the University of Rochester, institution compliant with the Public Health Service Policy on Humane Care and Use of Laboratory Animals (U.S. Department of Agriculture (USDA) registration number 21-R-019; Animal welfare Assurance Statement A-3292-01). Experimental protocols were approved by the University Committee on Animal Resources (UCAR).

### Murine collagen-induced arthritis

Studies were conducted at Washington Biotechnology, Inc., *in vivo* services Contract Research Organization (CRO) for drug development. The animals were maintained and treated in the facilities accredited by AAALAC in compliance with NIH and USDA standards (USDA registration number 51-R-031; Animal Welfare Assurance Statement A- 4192–01).

Male, 5–6 weeks old, DBA1/J mice (Jackson Laboratories) were immunized with 100 μg of bovine Type II collagen in CFA enhanced with 100 μg of heat-killed *Mycobacterium tuberculosis* strain H37RA (50 μl of collagen/CFA emulsion were injected subcutaneously at the base of the tail). Prophylactic treatment started on day 20 post initial immunization. On day 21 the mice were given a booster subcutaneous injection of bovine Type II collagen in Incomplete Freund Adjuvant (IFA). Therapeutic treatment started on day 30 post-initial immunization. The animals had been scored for macroscopic signs of arthritis 3 times weekly using a standardized scoring system (provided by Washington Biotechnology, Inc): 0, no visible signs of arthritis; 1, edema and/or erythema of 1 digit; 2, edema and/or erythema of 2 digits; 3, edema and/or erythema of more than 2 digits; and 4, severe arthritis of entire paw and digits. Arthritic Index (AI) was calculated by addition of individual paw scores. The maximum arthritic index that could be achieved in any given animal was 16.

For histological evaluation, all four limbs were removed and individually preserved in 20 volumes of 10% neutral buffered formalin. After decalcification in 5% formic acid for 2–3 days, tissues were trimmed, processed for paraffin embedding, sectioned at 8 μm and stained with toluidine blue. Paws were embedded and sectioned in the frontal plane. When scoring paws or ankles from mice with lesions of type II collagen arthritis, severity of changes as well as number of individual joints affected were considered. When only 1–3 joints of the paws or ankles out of a possibility of numerous metacarpal/metatarsal/digit or tarsal/tibiotarsal joints were affected, an arbitrary assignment of a maximum score of 1, 2 or 3 for parameters below was given depending on severity of changes. If more than 3 joints were involved, the criteria below were applied to the most severely affected/majority of joints. The following parameters were taken into account when scoring the joints for severity of arthritis: inflammation, pannus formation, cartilage damage and bone damage. The following scoring system was used: 0, Normal; 1, Minimal changes; 2, Mild changes; 3, Moderate changes; 4, Marked changes; 5, Severe changes.

### ELISPOT

The membranes of the Millipore plates (Millipore; MSIPS4510) were activated with 35% ETOH prior to coating. The membranes were coated with 10 μg/ml of each of the following reagents diluted in DPBS: NP4-BSA; NP14-BSA, Imject BSA, Imject KLH. The plates were incubated at 4°C overnight. Plates were washed and blocked with 10% goat serum (Gibco) diluted in tissue culture medium (RPMI 1640/Penicillin-Streptomycin/L-Glutamine and 5x10^−5^ M 2-mercaptoethanol) for 2 h at room temperature. Mouse splenocytes were resuspended in tissue culture medium supplemented with 10% FBS, added at 2×10^6^ cells per well and cultured for 48 h at 37°C. The plates were developed using AEC substrate solution (BD; 551951) and sent for analysis to Cellular Technology, LTD.

### Statistical analysis

Statistical analysis was performed using Graphpad Prizm 5 software.
